# Synthesis of Common Arabic Handwritings to Aid Optical Character Recognition Research

**DOI:** 10.3390/s16030346

**Published:** 2016-03-11

**Authors:** Laslo Dinges, Ayoub Al-Hamadi, Moftah Elzobi, Sherif El-etriby

**Affiliations:** 1 Institute for Information Technology and Communications (IIKT), Otto-von-Guericke-University Magdeburg, D-39016 Magdeburg, Germany; Moftah.Elzobi@ovgu.de; 2 Faculty of Computers and Information, Menoufia University-MUFIC, Menoufia 32721, Egypt; sherif.el-etriby@ci.menofia.edu.eg; 3 Department of Computer, Umm Al-Qura University, Makkah 21421, Saudi Arabia

**Keywords:** Arabic handwritings, optical character recognition (OCR), handwriting synthesis, digital pens, word segmentation, word segmentation, feature extraction and analysis, Active Shape Model, recognition and interpretation, intelligent systems

## Abstract

Document analysis tasks such as pattern recognition, word spotting or segmentation, require comprehensive databases for training and validation. Not only variations in writing style but also the used list of words is of importance in the case that training samples should reflect the input of a specific area of application. However, generation of training samples is expensive in the sense of manpower and time, particularly if complete text pages including complex ground truth are required. This is why there is a lack of such databases, especially for Arabic, the second most popular language. However, Arabic handwriting recognition involves different preprocessing, segmentation and recognition methods. Each requires particular ground truth or samples to enable optimal training and validation, which are often not covered by the currently available databases. To overcome this issue, we propose a system that synthesizes Arabic handwritten words and text pages and generates corresponding detailed ground truth. We use these syntheses to validate a new, segmentation based system that recognizes handwritten Arabic words. We found that a modification of an Active Shape Model based character classifiers—that we proposed earlier—improves the word recognition accuracy. Further improvements are achieved, by using a vocabulary of the 50,000 most common Arabic words for error correction.

## 1. Introduction

Modern document analysis heavily depends on automated processes as pattern recognition or segmentation. These processes need to be trained using a database and validated using corresponding, suitable ground truth (GT), though. However, collecting handwriting samples is proved to be an error prone, labor- and time-expensive process [1]. Especially costly is the creation of ground truth, which is why databases often contain minimal ground truth that is only sufficient for a couple of specific problems.

There is still a need for the creation of new databases, such as the Persian PHOND-database, that contains isolated digits, isolated signs, multi-digit numbers, numerical strings, courtesy amounts, and postal codes [2]. Abdelaziz *et al*. present the new AltecOnDB [3], an Arabic word online database that contains 39,951 different vocables. However, mainly two free available databases exist in the case of Arabic offline handwritten words. The IFN/ENIT database [4,5] contains Tunisian town names, their GT define the under baselines and the Unicode of the handwritings. The IESK-arDB database, that we proposed in [6], contains international town names and common terms including GT for segmentation. Since both databases are limited by the number of samples and words and contain single words or small sentences only, we believe automatized generation of databases customized for specific research is a helpful complement. Advantages are that samples can be created easily and quickly for any word or text at any time, moreover detailed GT are created simultaneously. This is one reason that Arabic Handwriting synthesis recently gained more and more interest. Furthermore, advanced handwriting synthesis enables to control specific features of the samples, so the robustness of a method against these features can be validated.

We built a User Interface (UI) based synthesis system that turns UTF8-coded Arabic texts or lists of words into corresponding databases of artificial handwritings. Ground Truth are automatically generated XML-files that cover basic data as original Unicode, lower baseline and the location of script components (Pieces of Arabic Word, (PAWs), letters and diacritics) and additionally data as ArabTeX transliteration and letter trajectories. The system is capable of generating realistic syntheses of words, text lines and complete (single column) text pages.

The main contributions of this paper are:

- Generation of synthetic Arabic handwritings, including Arabic pseudo texts.
- Introduction of a new, segmentation based system of automatic Arabic handwritten word recognition.
- Validation of that system, by using synthesized word databases.

### 1.1. Arabic Script

The system and all its modules, which can be used for different image processing task, shall be tested on the example of Arabic Handwriting synthesis and recognition. The Arabic script has some special characteristics so that synthesis- or OCR-approaches for Latin script will not succeed without major modifications [7]. Important aspects of Arabic script are:

1. Arabic is written from right to left.
2. There are 28 letters (Characters) in the Arabic alphabet, whose shapes are sensitive to their form (isolated, begin, middle and end), see Table 1.
3. Only six characters can be in the isolated- or end-form, which splits a word into two or more parts, the PAWs. They consist of the main body (connected component) and related diacritics (dots), supplements like Hamza (أ). In case of handwriting, the ascenders of the letters Kaf (ك), Taa (ط) or Dha(ظ) can also be written as fragments.
4. Arabic is semi-cursive: within a PAW, letters are joined to each other, whether handwritten or printed.
5. Very often PAWs overlap each other, especially in handwritings.
6. Sometimes one letter is written beneath its predecessor, like Lam-Ya (ي) or Lam-Mim (لم), or it almost vanish away when are in middle form, like Lam-Mim-Mim (لمم) (unlike the middle letter of Kaf-Mim-Mim (كمم) ). Hence, in addition to the four basic forms, there are also special forms, which can be seen as exceptions. Additionally, there are a few ligatures, which are two following letters, that build a completely new character like LamAlif(ﻵ).
7. Some letters like Tha (ث), Ya (ي) or Jim (ج) have one to three dots above, under or within their “body”.
8. Some letters like Ba (ب), Ta (ت), Tha (ث) only differ because of these dots.

### 1.2. Related Works

In the literature, research addressing the issue of synthetic text generation can be classified into two main categories. Top-down approaches are typically based on physical models, that simulate the writing process itself [8,9]. Therefore, script trajectories are seen as a result of character key points, the writing speed, character size and inertia, which finally leads to the curvature of handwritings. These approaches are focused more on the physical aspects of writing than the actual synthesis outcome [10].

Bottom-up approaches, on the contrary, model the shape (and possibly texture) of handwritings itself. Hence bottom-up approaches are preferred in the context of image processing tasks as segmentation or handwriting recognition. Bottom-up approaches can be further categorized into generation of new samples of the same level, and concatenation to more complex outcomes, such as words that are composed of characters or glyphs [11,12]. Some synthesis approaches are restricted to one technique, however, a higher synthesis variation and flexibility can be achieved by combining both. A common generation technique is data perturbation, which is performed by adding noise to online or offline samples [13]. Another generation technique is sample fusion, that blends two or a couple of samples to produce new hybrid ones [14,15]. A better statistical relevance can be achieved using model-based generation [16,17]. This initially requires the creation of deformable models from sufficient samples—usually on character level—from which new character representations are generated.

Examples for deformable models are Active Shape Models (ASMs) and novel Active Shape Structural Models (ASSMs), which are used for generating variances of simple drawings and signatures [18]. ASMs are also applied for the classification of Chinese letters [19].

Concatenation of handwriting samples to units of higher levels can be done without connecting the samples in case of Latin-based scripts [11], proper simulation of cursive handwriting requires at least partially connection tough. For concatenation, there are approaches that connect offline samples directly [20], and those who use polynomial- [21], spline- [22]. or probabilistic-models [23]. Due to the semi-cursive style, connecting is mandatory in case of Arabic script.

Systems using the described techniques to synthesize handwritings have been built for different scripts and purposes. Wang *et al.* [24] proposed a learning based approach to synthesize cursive handwriting by combining shape and physical models. Thomas *et al.* [25] have proposed synthetic handwriting method, for generation of CAPTCHAs’ (completely automated public Turing test to tell computers and humans apart) text-lines. Gaur *et. al* synthesized handwritten Hindi, Bengali and Telugu numerals and Hindi words for character and word recognition [26]. Multiple Latin specific approaches that are based on polynomial merging functions and Bezier curves have been documented in [22].

In contrast to word synthesis, little research has been done concerning text line, paragraph or document synthesis. If synthesis of multiple text lines is considered at all, it is typically modeled as horizontal baselines that may be influenced by noise [27].

As for the problem of automatic synthesis of offline handwritten Arabic text—to the best of our knowledge—Elarian *et al.* [4,5] wrote the first published research work addressing this problem so far. They propose a straightforward approach of composing arbitrary Arabic words. The approach starts by generating a finite set of letter images from two different writers, manually segmented from IFN/ENIT database, and then two kinds of simple features (width- and direction-feature) are extracted, so they can be used later as a metrics in the concatenation step. Saabni S. and El-Sana J. proposed a system to synthesize Pieces of Arabic Words (PAW) (without diacritics) [28] from online samples. We proposed a system to generate Arabic letter shapes by ASMs built from offline samples. Subsequently, we developed an approach to render images of Arabic handwritten words, concatenating samples based on online glyphs and using transformations on word level as optional generation step [29].

#### Handwriting Recognition

Handwriting recognition can be assigned to three main categories respective the use of segmentation. The first category contains all approaches that completely ignore the segmentation, such methods called "holistic" based [30,31] (Latin) [1], (Arabic). Under the second category fall all approaches that apply an over-segmentation on the PAW, and then a margining strategy is followed in order to detect the optimal margining path [32,33]. As an example for those approaches, Ding and Hailong [34] proposed an approach, in which a tentative over-segmentation is performed on PAWs, the result is what they called "graphemes", the approach differentiates among three types of graphemes namely (main, above, and under-grapheme). The segmentation decisions are confirmed by the recognition results of the merged neighboring graphemes; if recognition failed another merge will be tried until successful recognition. Also, an Hidden Markov Model (HMM) can be trained to handle segmentation [35]. The disadvantages of such approaches are the possibility of sequence errors and classification faults, as a result of the shape similarity between letters and fragments of letters. The third category is what is called "explicit segmentation", in which the exact border of each character in PAW is to be found. The main features often used to identify the character’s border are minima’s points near or above the baseline. Shaik und Ahmed [36] proposed an approach that used some heuristic rules calculated upon the vertical histogram of the word’s image. Though authors claim successfulness of their approach with printed text, they report failures cases when PAW contains problematic letters like Sin (س).

Our segmentation approach can also be categorized under this last category, since we are using topological features to identify the character border. The main problem with this category of segmentation is the varying of shape and topology within the single classes of handwritten Arabic letters. The feature extraction of the segmented letters [37] is the second important step of any recognition system. A common method to recognize words using explicit segmentation are Support Vector Machines (SVMs) [38,39].

Features for character recognition are often gradient or moment based and calculated for sub-images, resulting in feature vector with a length of around 100. Shanbehzadeh *et al.* [37] used moment like features, which are calculated for all columns of cells resulting in a feature vector of the length 34. In the approach of Parkins and Nandi [40] a histogram of the 8-neighborhood is used to extract features from each cell. Chergui *et. al* extract SIFT key points from 5 cells performing recognition by key point matching based on the Euclidean distance [41].

A straightforward, well-known approach of interpreting such feature vectors are *k*-Nearest Neighbor (*k*-NN) classifiers [42]. There is no need to train *k*-NNs, but the distance of the features of a sample to all training samples has to be computed, in order to classify the sample as those class, that is mostly represented in the *k* closest training samples. As a matter of fact, the classification step is quite costly if many training samples are used. Hence, clustering techniques may be used to reduce the number of comparisons [43]. SVMs allow classification by separating the feature space by hyperplanes [44]. A regular SVM separates one specific class from the rest. However, solutions—as LIBSVM—that handle multiple classes, might be more effective. Good character recognition results were also achieved by Artificial Neural Networks (ANNs). Cireşan *et al.* use deep convolutional neural networks (DNNs) to recognize digits and characters [45]. Unlike common ANNs as Multi-Layer-Perceptrons (MLPs), the design of DNNs is close to a biological model, and therefore, complex. Hence, the DNNs are optimized for modern GPUs, to speed up training. DNNs outperforms other ANN based character classifiers and can further be used for general, image-based classification problems [45].

Isolated-word error correction basically involves the often successively steps of error detection, generation of candidate corrections and their ranking [46]. Error detection is often solved by N-Grams, which checks whether a word shows any invalid combinations of *n* following characters. Comparing the word with a vocabulary—that also defines the candidate corrections—is a common, too. The ranking is often performed by a probabilistic estimation of the likelihood of the correction. However, the decision of the final correction is sometimes done by the user.

**Figure 1:**
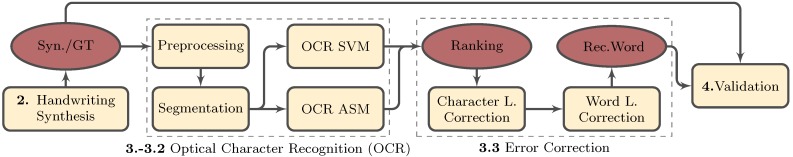
Overview of the proposed approach.

The rest of the paper is organized as follows. In Section 2, handwriting synthesis is discussed, as shown in Figure 1. We outline necessary data acquisition steps including the basically mathematical background of ASMs. Then, we summarize our methods to synthesize Arabic Handwritings and propose the resulting extension of our IESK-arDB database. In Section 3, a segmentation based approach for recognition of handwritten Arabic words is proposed. Thereafter, experimental results are discussed in Section 4, where we use synthetic databases to validate word recognition. Conclusion and future work are presented in Section 5.

## 2. Synthesizing Arabic Handwriting Databases

We built a system for Arabic handwriting synthesis, that can be used either to generate a couple of handwriting images, PDFs or LaTeX files directly, or to generate a complete word or paragraph database.

The first way is suitable for preview purpose in order to control the effect of different settings. Therefore, Arabic Unicode can be typed directly into the user interface or loaded from a file. When a setting is built, checked and saved, the second way allows to create synthetic databases as those we used for the experiments in Section 4. Arabic words or texts are loaded from UTF8-coded text files, then databases can be created automatically. Multiple settings can be used, in order to increase diversity. Ground Truth (GT) are created automatically and contain the Unicode, all bounding boxes of the PAW, letters and diacritics and furthermore text line affiliations and the trajectories of all letters.

The methods, which are used to synthesize and render Arabic handwritings, are described in the following sections, starting with the data acquisition step.

### 2.1. Data Acquisition using Infrared and Ultrasonic Sensors

**Figure 2:**
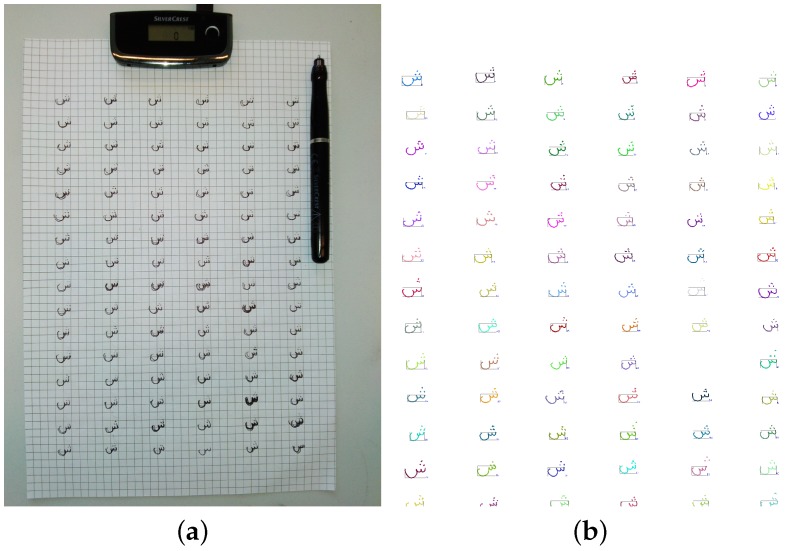
Online data acquisition. (**a**) Structure of data acquisition using the digital pen. (**b**) Visualization of the automatically extracted letter trajectories.

To synthesize Arabic handwritten words from glyphs, a sufficient amount of glyph samples has to be acquired first. In our case, trajectories of single letters and their connections (Kashidas) are used as glyphs. The glyphs are acquired with online techniques since relevant information can be extracted more efficiently from trajectories than images. In fact, building suitable glyphs without comprehensive manual labeling fails in case of about 50% of the character classes, in case that offline samples are used . Nevertheless, we are mainly interested in synthesizing offline handwriting samples (images), supplemented by optional trajectories within the ground truth (GT). Hence, we decided to use digital online pens for data acquisition, for they can be applied as conventional biros in contrast to digital tablets, that enforce crude handwriting style. We acquired all glyph samples by a SilverCrest DGP1000 digital pen which is using stereo infrared and ultrasonic sensors. These sensors are located at the corners of a receiver that has to be fixed on top of the writing surface (typically DinA4 paper sheets on a solid blotting pad), as shown in Figure 2a. The receiver can be connected to a PC via USB 2.0, which enables monitoring of received trajectories in real time. We found that these trajectories reflect the original handwritings well. However, occasional distortions are possible, if the receiver is not fixed properly.

Fifty or more samples per writer are taken from over hundred letter classes (28,046 samples altogether) to built an online character database. To minimize manual effort and allow an easy extension, this database is completely automatically generated from raw data. Raw data are trajectories $\overset{´}{X}\in R^{m\times 2}$ for each stroke within a virtual ISO A4 page. That page has a spatial resolution of 1000 dpi and 58 rps (reports per second), so there are constant timestamps $\overset{´}{▵t}=t_{i+1}-t_{i}=0.0172\;s$ between neighbored $\left({\overset{´}{x}}_{i,1},{\overset{´}{x}}_{i,2}\right)\in \overset{´}{X}$. The generated database contains the trajectories and an image representation for each Arabic letter class, as well as the resulting ASMs, which are described in the next Section. Digits and special characters are not included, but might be added in future work.

### 2.2. Active Shape Models

Active Shape Models (ASMs) represent the approximated shape of object classes statistically.

An ASM is created by first calculating the expected value $\bar{x}$ and the covariance matrix S:

(1) $$\bar{x}=\frac{\sum_{i=1}^{r}x_{i}}{r},\;S=\frac{1}{2r-1}\sum_{i=1}^{r}{\left(x_{i}-\bar{x}\right)}^{}{\left(x_{i}-\bar{x}\right)}^{T}$$

where *r* is the number of training samples. Then Eigenvalues $\lambda_{i}$ and Eigenvectors $e_{i}$ of S are calculated. Now any number of new vectors u—that represent the ASM—can be created by linear combination:

(2) $$u=\bar{x}+\sum_{j=1}^{r}c_{j}e_{j}\;|c_{j}\in \left[-2{\sqrt{\lambda }}_{j},2{\sqrt{\lambda }}_{j}\right]$$

**Figure 3:**
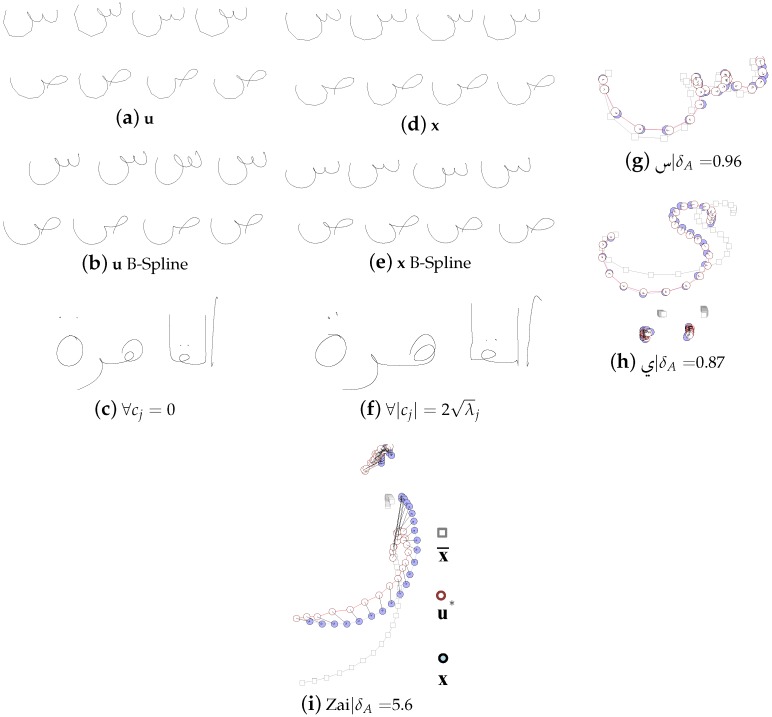
Building ASMs. (**a,b,d,e**) Examples for ASM representations u (where $\sum |c_{j}|\approx \sum 1.6{\sqrt{\lambda }}_{j}$, see Equation (2) ) and original letter samples **x**. B-Spline interpolation with 5 steps has been applied on (**b**) and (**e**). (**c**) and (**f**) show examples of u that are composed to words. Examples of ASM representations $u^{*}$ that fit to samples x with the deviation $\delta_{A}$ are shown in (**g**–**i**).

To ease the following steps, we rearrange $\overset{´}{u}$ to get polygons $U\in R^{n\times 2}$. Some examples of x and u are shown in Figure 3. To compute over 100 ASMs per writer is very time consuming, especially if many samples and interpolation points are used. Hence, we saved them and the original samples as XML-files which can be loaded quickly by the synthesizing system or other applications.

### 2.3. Word Sample Synthesis

The basic idea of Arabic handwriting synthesis from Unicode is to select glyphs with proper shapes (isolated, initial, end or middle form) and connect them subsequently to build Pieces of Arabic Words (PAWs), words and texts from which images or vector graphics are rendered.

To achieve variability in letter and word shape, ASM generated samples are used and modified by optional affine transformations as scaling, shearing, and rotation, in order to simulate variation in slant and skew or stretched letters.

**Figure 4:**
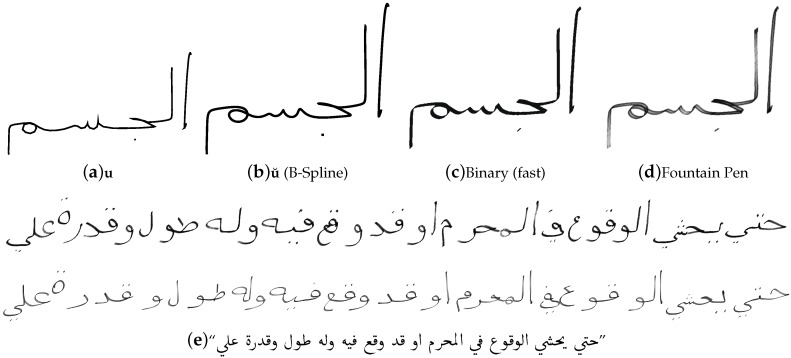
From the Polygon u to a synthetic image that simulates a feather like writing instrument. (**a–d**) Visualization of the used methods. (**e**) Two syntheses of an Arabic Unicode text line demonstrate the effect.

After the concatenation step, we use B-Spline interpolation to improve the trajectories u, as shown in Figure 4a. This step is optional, but might be necessary in some cases, since the polygonal letter representation does not look natural for vector graphics or images with a scaling factor >1. As shown in Figure 4b, the resulting trajectory $\overset{˘}{u}$ looks more smooth and natural.

The method, which is used to render the actual synthetic sample images, includes two steps. Firstly the computation of pixels, which are effected by a virtual pen, that follows the letter trajectories. Secondly the generation of ergodic pigmentation maps and filters, which define the pen texture. Further details of our synthesis approach are given in [29].

For rendering IESK-arDB${}_{\mathrm{SynWords}}$ and IESK-arDB${}_{\mathrm{SynText}}$, we used an improved approach, that simulates the features of (binarized) fountain pens or feathers writings, as shown in Figure 4c. This is important, particularly when historical handwritings are synthesized, and achieves furthermore a higher variability of the synthesis. Moreover, the effect of semi-opaque pigmentation caused by increased writing speed can be achieved, that we describe in [29]. An example is shown in Figure 4d.

### 2.4. Generation of Pseudo Texts

**Figure 5:**
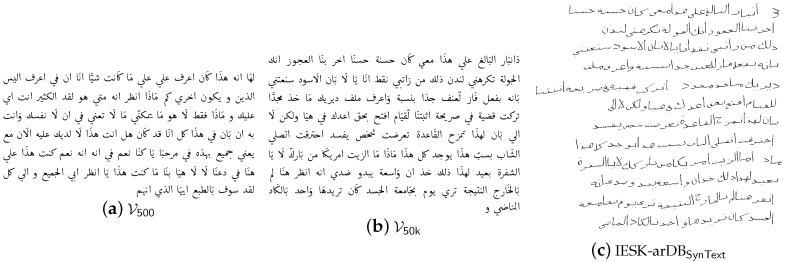
Examples for Arabic pseudo text, which is Unicode text generated by linking words of a vocabulary V. (**a**) 500 different, (**b**) 50,000 most common Arabic words are used. (**c**) A text image, that was synthesized from a pseudo text page sample.

Recently, the focus of Arabic handwriting research has changed from character or word recognition to more complex document analysis issues, that address the interpretation or recognition of complete documents. Hence, we investigate methods for text page syntheses. In this regard, the accurate simulation of the lower baselines is crucial, as we revealed in [29]. An example is shown in Figure 5c.

Concerning text recognition, also the used words have a strong influence on the outcome, since common texts show often relatively small words, compared to sets of words that are used for a specific problem as letter head recognition. For this reason, we use the vocabulary of the 50,000 most common Arabic words $V_{50k}$ and their frequencies $n(w_{i})$ within Unicode texts (available at [47]). As a consequence, pseudo text can be built by stringing a couple of $w_{i}\in V_{50k}$. To respect the word distribution, we simply compute the a priori probability of occurrence of a word $w_{i}$:

(3) $$P\left(w_{i}\right)=\frac{n(w_{i})}{\sum n(w_{i})}$$

Subsequently, wheel of fortune technique is used to choose iteratively a word according to the probability $P(w_{i})$. This way, pseudo text as shown in Figure 5 are built. As one can see, Figure 5a exhibits more small words than Figure 5b. However, even a text that is based on a large vocabulary contains typically more small words, and larger words are often split into several Pieces of Arabic Words (PAW). A sample of a synthesized text using a moderate modulation of baselines is shown in Figure 5c. In this paper however—since we do not concentrate on segmentation of texts into lines and words—databases of word samples are used instead, that are discussed in the next section.

### 2.5. Extension of the IESK-arDB by Synthesised Samples

Recently, the proposed synthesis system is not freely available. However, we extended our existing IESK-arDB -database (www.iesk-ardb.ovgu.de by synthesized text pages (IESK-arDB${}_{\mathrm{SynText}}$) and words (IESK-arDB${}_{\mathrm{SynWords}}$).

Regarding this paper, IESK-arDB${}_{\mathrm{SynWords}}$ is of essential interest. It contains 20,000 words synthesis using pseudo-text, that is based on $V_{5k}\subset V_{50k}$—the vocabulary of the 5000 most common Arabic words—that is also used for the main experiment in Section 4. Each sample of IESK-arDB${}_{\mathrm{SynWords}}$ is a png-image and a corresponding XML-file, that contains the ground truth (GT) (including letter trajectories, to enable eventual validation of online methods as well).

**Figure 6:**
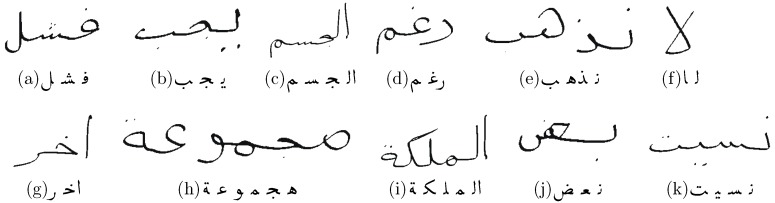
Samples of synthesized Arabic Words from the IESK-arDB${}_{\mathrm{SynWords}}$ database. (**a**) فشل, (**b**) يجب, (**c**) ﺍلجسم, (**d**) رغم, (**e**) نذهب, (**f**) ﻵ, (**g**) ﺍخر, (**h**) هجموعة, (**i**) ﺍلملكة, (**j**) نعﺽ, (**k**) نسيت.

In the context of this paper, we are mainly interested in those features, which are related to a word $w_{i}$ itself (as the number of PAWs or the permutation of characters). Hence, all syntheses of IESK-arDB${}_{\mathrm{SynWords}}$ show only moderate deviations in kashida length, skew or slant or other features, that can be controlled by the current version of our synthesis system. That very version includes already strategies to avoid collision of text lines, neighbors words or PAWs. However, intersection between characters of the same PAW—as shown in Figure 6j—are only detected and noted within GT, but not solved. Such characteristics could even be observed in real handwritings, hence we do not exclude these samples from our experiments, even though they often prevent correct segmentation. Furthermore, we include words, which are especially hard to segment. This words contain the problematic characters (ص س ش ﺽ), as for example Figure 6k. Often, such words are not included in the test set for segmentation. However, about 25% of $V_{50k}$ contain at least one of the problematic characters, which is why we expect that most real-world problems can not be solved excluding these words from the used vocabulary.

One of the main applications of synthetic handwritings is to aid automatic handwriting recognition research. In this sense, we propose an approach for word recognition in the next section, that is validated on IESK-arDB${}_{\mathrm{SynWords}}$ in the following experimental section.

## 3. Segmentation based Recognition of Handwritten Arabic Words

Recognition of Arabic handwritings is still challenging, and—due to the lack of suitable databases—it is a proper method to evaluate the utility of the proposed synthesis approach. In this paper, we focus on handwritings that reflect a general Arabic vocabulary.

The first step of the proposed word recognition approach is to segments Arabic handwritten words into character. These are then recognized by SVM or ASM based classifiers. Thereafter, *a priori* knowledge is used to correct possible segmentation or recognition errors. Therefore, we use the synthesized word databases, which allow validating segmentation and character recognition. At the same time, the general usage of IESK-arDB${}_{\mathrm{SynWords}}$ to aid automatic word recognition—especially in the context of handwritings, that reflecting average Arabic vocabulary—can be evaluated.

### 3.1. Segmentation

Our segmentation method is described in [6]. It is based on topological features and a set of rules, that reduces all candidates to a final set of points, which divide two neighbored letters. In contrast to other approaches, candidates are not minima, that indicate the middle of a Kashida, but typically the following branch point.

### 3.2. Character Recognition

After the word image has been segmented into single characters, the next step is to classify these characters. To enable further steps, as a combination of different classifiers or rejection of character segments, we rank all class candidates first. We tested Support Vector Machines (SVMs) and Active Shape Models (ASMs). In the following, their functionality, advantages and disadvantages shall be discussed. In the context of word recognition, some modifications of the samples, which are used for the training of the ASMs and SVMs based classifiers are required. In [48] we split all samples randomly in training and test samples, which is a common procedure when validating character recognition systems. Since handwritings are typically written by unknown writers, we train the ASMs as well as the SVMs again, ensuring that training and test set contain no samples of the same writer.

#### 3.2.1. Decision Trees

In contrast to the English Alphabet, some Arabic characters as (ج ح خ) or (ب ت ث) have identical main bodies. Hence, the only feature to differentiate between them is the number and position of dots. We detect these feature, as well as the existence of loops as an input of the decision tree. Due to the fact, that Arabic character shapes are heavily depending on the character form, each form requires its own sub decision tree. We build 10 character groups for each form. Some characters might appear in handwritings with or without a loop, typically in the **m**iddle form. Furthermore, even groups of two dots can be written as a single one. Hence, some characters occur in more than one character group. This is why we saved automatically generated character groups as XML, allowing manual monitoring and subsequent modifications. Such modification will require training all SVMs again, the ASMs however, are independent.

The following classification methods require the ID of the character group, where character x belongs to. Since diacritic marks might be misplaced in the case of handwritten words, there is a chance, that the wrong character group has been selected. This is one reason, why character classification results as part of word recognition are worse compared to pure character recognition (see Section 4).

#### 3.2.2. Support Vector Machines

Support Vector Machines (SVMs) are state of the art classifiers, which are easy to train and allow fast and reliable classification. They expect a normalized feature vector f, that we compute from the raw features $f_{i}^{\prime }$:

(4) $$f_{i}=\frac{f_{i}^{\prime }-{\bar{f}}_{i}^{\prime }}{\sigma_{f_{i}^{\prime }}}$$

We use SVMs with a nonlinear radial basic function (RBF) kernel, choosing the LIBSVM (A Library for Support Vector Machines) implementation that allows getting a ranking of all class candidates. RBF based SVMs require optimization of the parameter *C* and *γ* for each SVM, in order to avoid over or under fitting. Since many Arabic characters differ only or mainly because of their diacritic marks or loops, we build separate SVMs for all character groups, as discussed above. We also trained one-vs-all SVMs for all character classes in order to avoid, that SVMs have to be retrained when character groups were changed. However, we drop the latter approach, since a lower accuracy is achieved in this case.

For all SVMs, we keep the samples of one writer for testing and use the rest for training. Parameter *C* and *γ* are optimized for each SVM, using grid search and cross-validation, in order to avoid over- or under-fitting. Nevertheless, even though train and test samples are from different writers, problems might occur when a character, that is a result of the proposed segmentation, have to be classified. This is due to the disability of training as well as the test samples, to reflect all features of segmented characters, as possible fragmentation of neighbored characters, normalization faults, or excessively long or short Kashidas.

In [48], we tested the different state of the art feature of [37,40]. Using SVMs we achieved good results (95.4%±0.3% and 97.14%±0.06%), which are similar to our ASM based approach (97.69%±0.31%), while *k*-NN achieved less recognition accuracy (92.3%±0.06% and 55.6%±0.6%). Since the features of [37,40] are extracted from the normalized image, we add the aspect ratio as an additional feature. Combining more and more feature sets and reducing the feature vector length by Principle Component Analysis (PCA), improvement of accuracy is realizable while keeping the advantage of fast SVM classification. As a matter of fact, SVMs have been our favored classifier candidate for segmentation based recognition of Arabic Handwritings. Nevertheless, we will show in the experimental section, that SVMs—integrated into a word recognition system—perform worse than our proposed ASM based classification approach.

#### 3.2.3. Active Shape Models

ASMs represent the shape of character classes and are useful regarding generation of unique handwriting syntheses, as stated in Section 2.2. A common application of ASMs is also localization or tracking of specific objects or body limbs. This requires a proper initialization as well as the iterative process of adapting the ASMs to the target. In our case, however, a proper initialization is known, but not the character class. Hence, the ASMs of all candidate classes are initialized and fitted to the character image, to measure the similarity of sample and ASM. This way, ASMs can be used to rank all corresponding candidate classes, similar to one-vs-all SVMs.

Depending on the number of iterations, ASM based classification has relatively high computational costs. On the other hand, however, they show a dynamic behavior and enable a more directly comparison with test samples, than other classifiers. Even though we do not use the same ASMs for classification and synthesis, the same procedure as described in the last sections is used for their generation, and representations u are generated according to Equation (2).

##### Implementation

**Figure 7:**
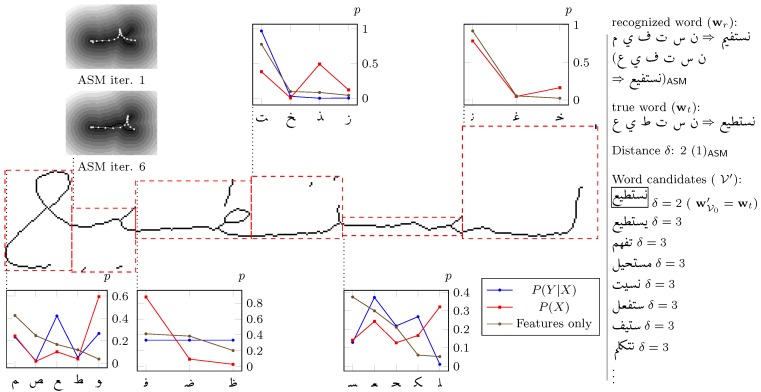
Example output of our word recognition system that uses the proposed segmentation method and SVM/ASM based classification. The diagrams show the SVM based ranking and the a priori knowledge for the single characters. As one can see on the right, the errors of the recognized word $w_{r}$ could be corrected by comparison with the sorted vocabulary $V_{}^{\prime }$.

In contrast to SVMs, k-NN or Gaussian Mixture Models, ASMs are not trained on any statistical feature vectors. In fact, ASMs reflect the shape of all samples from which they are built, as discussed in Section 2.2. To use ASMs for classification, suitable representations $u_{i}$ are created for each iteration *i* and compared with the sample x, which is why ASM based classification is similar to clustered k-NN classifiers. However, due to the dynamic behavior of ASMs, new $u_{i}$ are created for each classification. As a consequence, it would be necessary to calculate features for $u_{i}$ for each classification process again. Hence, statistical feature vectors f as proposed in [40] would not be efficient for ASM based classification. Instead of computing f for all test samples x and ASM representations $u_{i}$, a chamfer transformed image of x can be computed, as proposed by Shi *et. al* [19], and compared with u. After initialization, the current correlation of $u_{i}$ and x depends on the distances of the 25 coordinates of $u_{i}$ to the nearest edge of the sample. This is simply the value of the underlying chamfer image, as visualized at the upper left of Figure 7. This measure has low computational costs and is vital to fit the ASM to the sample by optimizing the used eigenvector weights $c_{j}$ of Equation (2). Costlier measures are used to compare x and $u_{i}$ in the case of the final iteration.

Initialization can cover normalization of aspect ratio (which has then to be used as an additional measure). As we describe in [48], up to 4 additional measures are used, mainly to reduce false positive matches. To improve the proposed method, also feature vectors could be used at this point, extracted from a normalized image of $u_{i}$, that can be generated by the proposed synthesis approach.

In this paper, we investigate offline handwriting recognition only. However, our ASMs based classification would be suitable for online classification as well, that would allow faster and more accurate fitting of the ASMs.

ASMs do not depend on their samples so strictly, as SVMs do. For instance, modification of the Kashida length or rotation is possible even during the classification process. This way, we reduced the right-hand Kashida of all ASMs in middle or end form, without the need of recomputing them or their corresponding training samples. As shown in Figure 7, the ASMs start with a moderate Kashida and adapt Kashida in the following iterations. Further parameters, as slant or rotation, can be activated at classification state. However, no further improvements are observed in this case, since IESK-arDB${}_{\mathrm{SynWords}}$ contains only moderate variations of those features, which can essentially be modulated by $c_{j}$.

An advantage of the proposed ASM based classifier compared to clustered k-NN classifier is, that not just a fixed set of training samples are compared with x. Instead, the optimization of an ASM involves as few representations u as possible.

##### Optimization

We investigated three different optimization techniques: Gradient Descent (GD), Genetic Algorithm and Simulating Annealing. Since the origin of the optimization problem is the expected character shape $\bar{x}$ of a handwritten character, Gradient Descent, that is sensitive to a proper origin, gives best results [48]. GD requires twice as many u to compute the gradient for one iteration, as the number of degrees of freedom (which are the used eigenvectors and variations in Kashida *etc.*). We found—in the case of pure character classification—that about 60 representation u (using the first 6 of 25 eigenvectors) are sufficient to achieve almost maximal classification accuracy (for GD and the other two optimization methods). However, the ASM-based approach is still quite costly. On the other hand, it is slightly more accurate than *k*-NN and SVM-based classification, which use sets of more complex features [48].

With the intention to improve accuracy, we built separate ASMs for each writer in order to combine the rankings of multiple classifiers, using the average and standard distribution. However, the costs of classification gain with the number of ASMs. More importantly, in contrary to expectations, even better results could be achieved using the same training samples to built a single ASM, which is therefore used for the experiments in Section 4.

### 3.3. Word Recognition

Our word recognition approach combines the proposed segmentation and character classification methods in a system, that automatically recognizes digital images of single handwritten Arabic words. It also includes error correction, that is described in the following section.

From the ASM or SVM based rankings of all segmented words, we derive a sequence of characters $w_{r}$, that is the recognized word before error correction, where $w_{r}$ has the class of the first entry of the corresponding ranking. If segmentation and character classification are both correct, $w_{r}$ is a valid Arabic word (given, that the handwriting contains no mistakes or foreign words). Otherwise, $w_{r}$ deviates from the real word $w_{t}$ with the Levenstein distance *δ*. An example is shown in Figure 7.

In the next section, we will propose strategies to recognize the handwritten word even in case that $w_{r}\neq w_{t}$.

#### 3.3.1. Error Correction

Even if the Accuracy of character classification is quite high, the recognized character probably deviates from the real word. If $P(w_{r}=w_{t})$ is the probability, that a character $w_{}$ is classified correctly, than the probability, that a word $w_{t}$ of size *n* is recognized correctly, is $\prod_{1}^{n}P\left(w_{r_{i}}=w_{t_{i}}\right)$. Furthermore, segmentation errors are likely, which means, that the length of $w_{t}$ and $w_{r}$ might differ, due to missing or additional segmentations. This might increase *δ* by 2, in case of character fusion or splitting. Even minor faults, as imperfect positions of segmentations, can increase *δ* and decrease recognition accuracy. Hence, a correction of such errors should be performed. In the following, we investigate correction on character level, which is based on statistical distribution of the characters, and correction on the word level, which is based on a comprehensive vocabulary of expected words.

##### Character Level

Depending on the similarity between classes of a character group and the grade of degeneration of a handwritten sample x, a reliable feature based ranking might be impossible in some cases. Similar characters are for example (د ر ل ك). If the features extracted from x are ambiguous, it could be useful to choose the character class, that is statistically more likely. In such cases, a poor contrast of the first and the second entry of a ranking is expected, as for example shown in the third diagram of Figure 7 (character ط). To support the feature based ranking in this way, we calculated the a priori probabilities P(X) and P(Y|X) from $V_{50k}$.

P(X) is the probability, that a character $w_{i}$ is of class *X*. If r is the vector of keys of the feature based ranking, than the statistically improved ranking $r_{A}$ can be computed by:

(5) $$r_{i_{A}}=\left(1-\lambda_{A}\right)r_{i}+\lambda_{A}P\left(X\right)$$

where $\lambda_{A}$ needs to be optimized. The a priori probability varies from 0.5 for P(X=ﺍ) to 0.0007 for P(X=ق), and also depends on character form. If $\lambda_{A}\neq 0$, we use the calculated P(X) for $w_{0}$ and P(Y|X) (2-gram) for all following characters, where X,Y are character classes of the Arabic alphabet (ignoring the character form).

**Figure 8:**
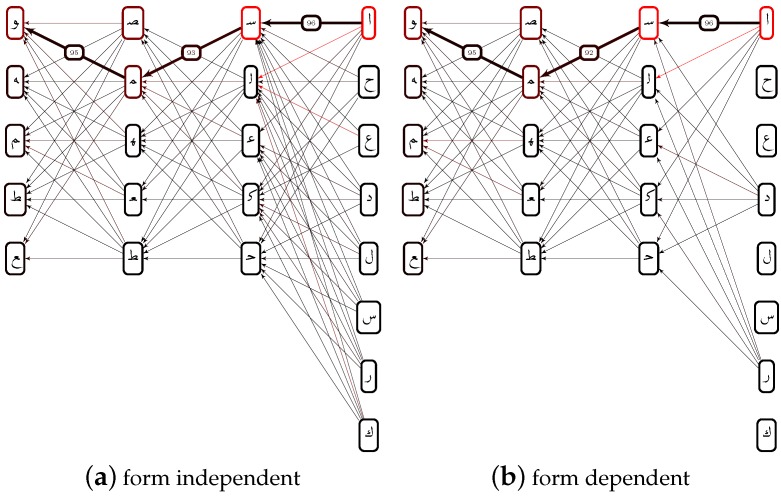
Error correction using a priori knowledge on character level. An error could be detected, since, according to the vocabulary $V_{50k}$, no ص can follows after س. Feature based rankings go top down and are indicated by the node color, a priori by the edge color (dark ∼ high costs).

P(Y|X) is the probability, that $w_{i}$ is of class *Y* in case that $w_{i-1}$ is of class *X*. However, if $w_{i-1}$ is not of class *X*, P(Y|X) would statistically even decrease the chance of correcting a misclassification of $w_{i}$. As a matter of fact, in contrast to P(X), the statistical usage of P(Y|X) depends on how reliably $w_{i-1}$ has been classified. This is, why we connect the rankings of all characters to a graph, as shown in Figure 8. This way, we can involve the likelihood, that $w_{i-1}$ is indeed of class *X*. Dijkstra algorithm is applied to find the path with minimal costs. In case of $w_{i}|i>0$, the cost of an edge X→Y are given by P(Y|X) and the classification ranking $r_{i}$ (that is the node, at the end of X→Y):

(6) $$X\to Y_{\mathrm{Costs}}\approx {\left(\left(1-\lambda_{A}\right)r_{i}+\lambda_{A}P\left(Y|X\right)\right)}^{-1}$$

For the first character $w_{0}$ we use $X\to Y_{\mathrm{Costs}}\approx r_{i_{A}}^{-1}$. As a consequence, the word $w_{A}$ is given by the path with minimal average $\bar{X\to Y_{\mathrm{Costs}}}$, as shown in Figure 8.

In contrast to error correction on the word level, this technique limits only the used language (as Arabic, Urdu, Farsi), but not the number of recognizable words. However, poor results are achieved when using ASM classifier, and even combined with SVMs, only moderate improvement could be achieved, as discussed in Section 4. Also the use of form dependent probabilities $P(Y,X_{i/e/m/b})$ could not improve results. Hence, error correction on word level—that we outline in the next section—is a vital addition.

##### Word Level

Error correction on word level is based on a given vocabulary V of valid words. In our experiments, the used vocabularies are subsets of the 50,000 most common Arabic words, as $V_{5k}$, that includes the first 5000 words. Since words, which do not appear in V can not be recognized, the definition of a comprehensive V or one that reflects the needs of specific circumstance is vital. In our experiments, all samples are included within V, so this paper does not cover rejection of words. However, long names or technical terms, that are typically not included in $V_{50k}$, will show little similarity with most words of V. Hence, rejection can be performed if *δ* exceeds a certain threshold.

Given the true word sample as a character sequence $w_{t}\in V$, the recognized character sequence $w_{r}$ is either identical with $w_{t}$ or differs because of one or multiple segmentation or recognition faults. In case of $w_{r}=w_{t}\Rightarrow w_{r}\in V,$ the direct recognition of the word has been successful. Otherwise, the most similar vocable $w_{V_{i}}≃w_{r}\;|w_{V_{i}}\in V$ have to be found, which fulfills

(7) $$\underset{\delta }{\arg}\;\min\left(\delta \left(w_{V_{i}},w_{r}\right)\right)\;|\delta \in N_{0}$$

We use Levenstein distance as measure *δ*, that has to be calculated for all $w_{V_{i}}$. Thereafter, $w_{V_{i}}$ are sorted increasingly according to *δ*, getting a list of candidate words $V_{}^{\prime }$, as shown in Figure 7. We choose $w_{V_{0}}^{\prime }$ as corrected word, even in case that the following $w_{V_{i}}^{\prime }$ have the same distance to $w_{r}$ (which is likely for huge $V_{}$ and small $w_{t}$). It may happen, that the vocabulary includes multiple declinations or conjugations of the same root. In such cases, the root word might be recognized, even if $w_{V_{0}}^{\prime }\neq w_{t}$. Hence, it might be also of interest, how often $w_{t}$ is under the first ten $w_{V_{i}}^{\prime }$.

Even if a vocable $w_{V_{i}}^{\prime }$ fulfill δ=1, it has probably not even the same word root as $w_{t}$, and multiple $w_{V_{i}}^{\prime }$ could show the same *δ*. This is especially true if $w_{t}$ and similar vocables $w_{V_{i}}^{\prime }$ are short (2–4 characters). In this case, many $w_{V_{i}}^{\prime }$ differ only in one character, and can be easily confused with each other. Many short vocables are also quite frequent. As a matter of fact, correction of $w_{r}$ when $w_{t}$ is one of these frequently used vocables, becomes especially challenging. To overcome this pitfall, we calculated the a priori probability of occurrence $P(w_{V_{i}}^{\prime })$ of the 50,000 most common Arabic words, to complement the measure *δ* by adding $P(w_{V_{i}}^{\prime })$. Since $P(w_{V_{i}}^{\prime })<1$ for vocabularies that contain more than one entry, the order of $w_{V_{i}}^{\prime }$ is only influenced within neighbored $w_{V_{i}}^{\prime }$ of the same *δ*. This doubles the effectiveness of error correction on the word level, in case that $V_{5k}$ is used. Even with $V_{50k}$, good results are achieved. New, application specific vocables, for which $P(w_{V_{i}}^{\prime })$ is not known, can still be added to the used vocabulary.

## 4. Experimental Results

In this section, we use databases of synthesized Arabic Handwritten words to validate the proposed word recognition system and its most vital components: segmentation and character recognition. Furthermore, we intend to investigate, how features of common texts influence the success of word recognition.

In contrast to word composing methods, which use permutations of original samples, our ASM based method allows computing any number of unique synthetic words, while the variation of the character shapes can be controlled continuously. This way, new databases to validate different applications—that may be related to a specific vocabulary (as town names, technical terms *etc.*) or to a general vocabulary, as we proposed in this paper—can be generated efficiently.

### 4.1. Segmentation

The synthetic samples, which are created by the proposed approach, are meant as training or testing data for different document analysis methods. To investigate, whether these syntheses can be used instead or additional to real handwriting samples, we created synthetic samples (png files + ground truth) of all words of the IESK-arDB database [6], that we call IESK-arDB${}_{\mathrm{Syn}}$ in the following.

The segmentation method, which is used for the following experiments, is described in [6]. It is based on topological features and a set of rules, that reduces all candidates to a final set of points $p_{d}$, which divide two neighbored characters. In contrast to other approaches, candidates are not minima, that indicate the middle of a Kashida, but typically the following branch point, that can even be quite close to the center of a character. This complicates validation since even a human is not always able to decide certainly, whether a segmentation is correct or not. In most cases, the GT of both IESK-arDB and IESK-arDB${}_{\mathrm{SynWords}}$ define $p_{d}$, that are located in the middle of the kashida between two words. Hence, some errors, which are detected by the automatic validation, might actually be still acceptable segmentations, even if detected $p_{d}$ differ clearly from GT. This is why automatic validation of segmentation only shows approximated behavior, and should be complemented by validation of character recognition and the complete word recognition system in the following sections.

As shown in Table 2, similar results are achieved for real (IESK-arDB) and synthetic data (IESK-arDB-Syn) using the proposed validation technique. However, the results are clearly better for $V_{5k}$, in particularly the rate of perfectly segmented words. A proper explanation is, that the 5000 most common Arabic words are in average shorter than IESK-arDB (which, on the other hand, complicates error correction). Segmentation errors are typically of two cases:

1. Oversegmentation, that splits a character into two
2. Undersegmentation, that fuses two characters into one

Hence, two aftereffects are expected in the following classification for each segmentation error. However, there are cases, where a segmentation is just imperfect, but still allows a correct classification. This is why the detected error *δ* only approximately correlates with the measured accuracy of segmentation and character recognition. Advanced experiments, that investigate the robustness of segmentation against different features of handwritings are discussed in [29].

### 4.2. Character Recognition

Segmentation is the most crucial step in segmentation based Arabic Handwriting recognition. However, the following character recognition is vital for a successful word recognition, too. In this context, robustness against minor segmentation or preprocessing faults is an especially valuable feature.

We proposed ASM- and SVM-based classification, both applied after the necessary reduction of the class candidates by a decision tree. We found, that both ASM- and SVM-based classification gives good results when splitting a character database in train and test set [48]. However, integrated into the word recognition system, ASMs perform better than SVMs.

#### 4.2.1. SVM based OCR

**Figure 9:**
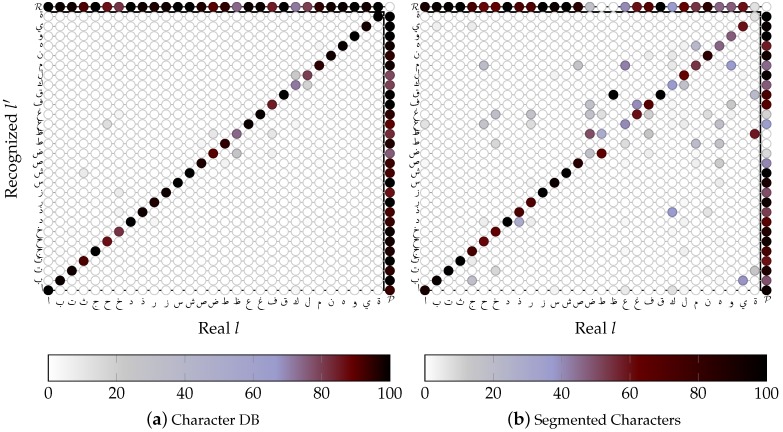
Confusion Matrix of writer independent Support Vector Machines (SVMs). (**a**) SVMs are trained and tested on a character database: average Precision $\bar{P}$ 94.2116%, average Recall $\bar{R}$ 94.2063%. (**b**) Synthetic words are segmented using GT, than each segmented character is classified using decision trees and SVMs ( $\bar{P}=64.716\%$, $\bar{R}=65.1279\%$).

As one can see in Figure 9, character recognition using SVMs is clearly less precise when segmented samples are used instead of a character database. Some of the confusions are caused by the decision tree, and will occur as well when using ASM. Nevertheless, many confusions are a result of segmentation, that changes the relation of the left and right-side Kashida. Note, that the number of samples are equal for Figure 9a, but depend on their class a priori probability of occurrence P(X) in case of Figure 9b, since they are taken from words of $V_{50k}$.

**Figure 10:**
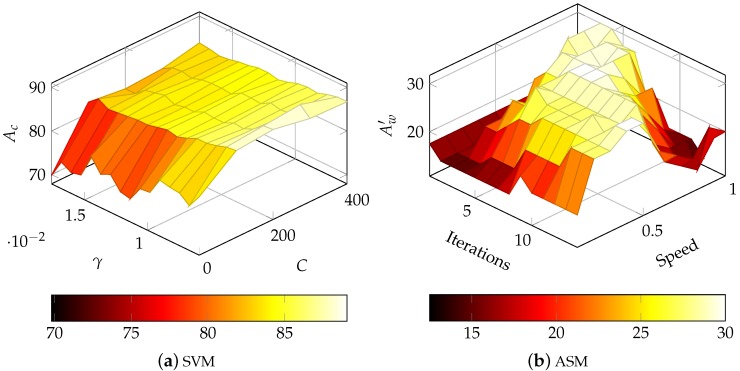
Optimisation of (**a**) SVM of the character group (ل ﺍ ح د ر س ع ك) in isolated form: RBF kernel is use, so *C* and *γ* have to be optimized. (**b**) ASM Classifier (during word recognition); The speed and the number of iterations of the Gradient Descent algorithm are optimized, which are most important the Accuracy and performance of ASM based classification.short

In Figure 10a, validation of the trained, writer independent SVMs is visualized. Each SVM is trained for one character group. On average, the writer independent SVMs achieve 94% accuracy (up to 99%, if training and test data contain samples of the same writers). Depending on the size and similarities of characters within a character group, an SVM performs above or below average; even though the (**b**egin) group (ﺿ ﻇ ﻓ) contains just three characters, its accuracy is the worst (46%).

##### Influence of the Character Form

As one can see in Table 3, the form of a character influences, how reliable it can be classified. Typically, characters have more features in **i**solated form, and furthermore no distracting Kashidas, that may vary in length, which explains the good accuracy compared to the **e**nd form. Surprisingly, even better results could be achieved in the case of **b**egin form. This might be explained by the fact, that six characters do not occur in **m**iddle or **b**egin form at all, which reduces the number of possible confusions. Arabic characters have minimalistic shapes in **m**iddle form, which is why they are harder to classify.

To built proper SVMs, we use the same number of samples for all character classes. However, in real case scenarios, it is crucial, whether classes with high resp. low precision and recall are especially rare or frequent. This is why we weighted the contributions of the single classes $C_{i}$ according to $P(X=C_{i})$, (values in brackets in Table 3). Especially in the case of isolated characters, the expected classification quality increases (probably since isolated ﺍ are easy to classify and very frequent). However, no extreme negative or positive influence could be observed in general.

#### 4.2.2. ASM-Based OCR

We give a detailed comparison of ASMs and SVMs used for character classification in [48]. The results of the proposed ASM character classifier are similar to those of the SVM classifier. However, in contrast to SVMs, the classification process can be manipulated even after building ASMs. Hence, we could optimize the most vital ASM classification parameter using the direct word recognition accuracy, as shown in Figure 10b. The number of iterations of the used gradient descent method and the speed following the calculated gradient is vital in oder to achieve proper classification results but also influence computational complexity.

In the next chapters we will show that word recognition benefits from ASM based classification.

### 4.3. Word Recognition

The percentage $A_{w}$ of correctly recognized words of a given handwritten text, is crucial in case of future work, where word recognition is integrated in high level document analysis applications. Even if $A_{w}$ is not sufficient to create a proper Unicode translation of the handwriting, it might still be possible to categorize the text. This is why the influence of the used character classifiers, the vocabulary size and the used error correction strategies on $A_{w}$ are the focus of this section.

**Figure 11:**
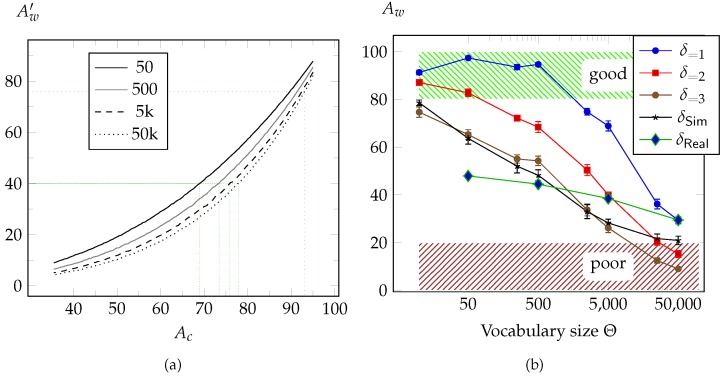
(**a**) Expected relation of the character recognition accuracy $A_{c}$ and direct word recognition accuracy $A_{w}^{\prime }$ for different vocabulary sizes; (**b**) Influence of the vocabulary size and error based Levenstein distance *δ* on the word accuracy $A_{w}$. Series $\delta_{\mathrm{Sim}}$ represents the simulated statistical error, based on the given segmentation and classification based errors. Series $\delta_{\mathrm{Real}}$ shows the behavior of the actual word recognition system.

Given a perfect segmentation result, the direct word recognition accuracy $A_{w}^{\prime }$ would depend on the character recognition accuracy $A_{c}$ and the average word length. This way, we can estimate the maximal $A_{w}^{\prime }$, that can possibly be achieved with a given $A_{c}$. On the other hand, a minimal $A_{c}$ can be determined, that is required in order to recognize $A_{w}^{\prime }\%$ of the words. As shown in Figure 11a, the expected outcome is influenced by another factor. Since frequent words are often shorter than less frequent, a better $A_{w}^{\prime }$ is achieved for $w_{V_{50}}$ compared to $w_{V_{50k}}$. However, even using GroundTruth (GT) for segmentation, $A_{w}^{\prime }=40\%$ is achieved only, using SVMs with an average $A_{c}$ of 94%. This deviation from the expectations is the result of faults, that are caused by segmentation or preprocessing. This is why even when GT are used, character classification in context of word recognition is less effective then predicted. On the other hand, additional faults occur even because GT are used, since PAW overlap solution—that would modify the word image—can not be applied in this case.

#### ASM *vs.* SVM

As mentioned above, the word recognition rate is clearly higher in case that ASMs are used for classification. Table 4 shows, that there is only a slight difference when GT are used for segmentation, but ASMs outperform SVMs when the proposed segmentation technique is used.

### 4.4. Error Correction

Due to variations in handwriting style and the similarity of some Arabic characters, the possibility to recognize a character correctly is limited, even for humans. In addition, imperfect segmentation increases this problem, as discussed before. Hence, strategies are required to correct this errors on character and word level.

We found, that error correction on character level achieves only minimal improvement, while those on word level—using a vocabulary of proper words to compare the recognized character sequence with—increases the percentage of recognized words clearly, as shown in Table 4. However, the number of words that can be recognized by the proposed system is reduced by the vocabulary size, that furthermore influence the accuracy of word correction, as shown in Figure 11b.

#### 4.4.1. Character Level Word Correction

Due to the fact, that the used SVMs are able to calculate a ranking of Likelihoods for all class candidates, error correction on character level using conditional probabilities P(Y|X) is more effective in combination with SVMs than ASMs.

The ASM based ranking is based on the geometrical deviation of the ASM and the sample x. As consequence, the contrast of the ranking is poor, and even with optimized $\lambda_{A}$ of 0.02, only minimal improvement could be achieved.

**Figure 12:**
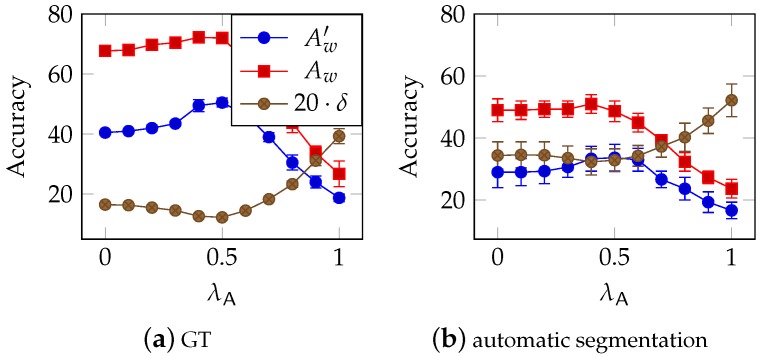
Optimization of the weight $\lambda_{A}$ of character level apriori information P(Y|X) on SVM based rankings (see Equation (6)). (**a**) As expected, best results are achieved for $\lambda_{A}$ of about 0.5. However, as shown in (**b**), there are only minimal improvement when segmentation errors occur, since they can not be corrected by P(Y|X).

In the case of SVMs, a distinct improvement is achieved for $\lambda_{A}\approx 0.5$. However, even though Figure 12a shows, that there is a moderate improvement of $A_{w}^{\prime }$ correcting character classification faults only, the effect is weakened when combined with word-level error correction. Furthermore, this technique is not suitable to correct segmentation errors, which is why the advantage is even less, as shown in Figure 12b. Hence, we conclude, that error correction on character level is not vital for the proposed word recognition system. The main problem is, that additional or missed characters can not be corrected that way. Moreover, this technique is applied after using the decision tree to reduce the number of class candidates, so classification errors, which depend on missing or wrong written diacritic marks, can not be corrected either.

In the following, we validate the proposed method to correct errors on word-level, focusing on the influence of Levenstein distance *δ* and vocabulary size Θ on the word recognition accuracy.

#### 4.4.2. Word Level Error Correction

The usefulness of the proposed error correction, that has been discussed in Section 3.3.1, is reflected by the word accuracy $A_{w}$, that defines, how many handwritten words are finally assigned to their corresponding vocable. However, $A_{w}$ depends on the used vocabulary as well as on the occurred segmentation and character recognition errors. These dependencies shall be investigated in the following experiments.

For cross-validation, we computed different sets $S_{\mathrm{ffi},\Theta }$, that contain modified versions $\dot{w_{V_{i}}}$ of randomly chosen vocables $w_{V_{i}}\in V_{\Theta }\subseteq V_{50k}$. By manipulating each word of $S_{\mathrm{ffi},\Theta }$, we ensure, that the Levenstein distance δ∈{1,2,3} to the original word—which is the simulated error—is always the same within a set. Samples of $\dot{w_{V_{i}}}$ are shown in Table 5. The first three series of Figure 11b show the sets $S_{1,\Theta }$, $S_{2,\Theta }$ and $S_{3,\Theta }$, and $A_{w}$ as a function of Θ. Errors are created by randomly adding, deleting or changing a single character. The experiment shows that the used error correction approach is capable of correcting one bit errors reliably for a vocabulary size of about 1000. Larger vocabularies, however, will clearly reduce $A_{w}$. Two or three-bit errors show a similar behavior; the error correction is frequently successful but still of use.

So far, the experiments show, that words, which contain 1–3 errors can often be corrected, at least in case that vocabularies of moderate size are used. However, we found the number of errors per word, which is caused by the proposed word recognition approach, have actually a strong variation. For the next series, we simulated the varying error $\delta_{\mathrm{Sim}}\geq 0$, that we expect from our word recognition system. We randomly add segmentation and then character recognition errors. Therefore, Gaussian distributions are used, which are computed from the measured over- and under-segmentation and the classification results. As one can see in Figure 11b, $A_{w}$ behaves similar to series three.

The next series shows results using the proposed word recognition approach itself. They obviously deviate from $\delta_{\mathrm{Sim}}$, especially regarding the influence of the vocabulary size. This means, that the measured distributions of segmentation and character recognition errors are not sufficient, to create a proper model of errors that are caused by the word recognition system. One reason is, that *δ* is not only influenced by variations of character shapes, but also by the permutation of characters and resulting number PAWs. Figure 11b shows, that, using a large vocabulary, good results are achieved for $\delta_{\mathrm{Real}}$ compared to the simulated series. However, reducing the vocabulary size is less effective than expected. Possible reasons could be, that some of the most frequent words are especially hard to segment.

#### 4.4.3. Computational Effort

Since word recognition might be used in a context that requires being real-time capable or handling a huge amount of words, not only the recognition accuracy but also the computational time per recognized word—caused by the used segmentation and classification methods—is of interest. All experiments were done on a machine with Intel(R) Core(TM)2 Quad CPU with 2.66 GHz and 4 GB RAM using Windows 7 (64 Bit). Currently, none of the proposed methods have been optimized for speed, only the database is loaded using parallel computing.

Table 6 shows the average time that is needed to load and recognize an Arabic word image (costly MATLAB functions are partially excluded). The relatively high variation is caused by the difference in word length and image size (which depends on latter but is also slightly influenced by the character classes and glyph shapes). SVM-based is clearly faster than ASM-based classification, that requires generation of ∼100 samples u for optimal results. If optional costly preprocessing steps (slant and skew correction) are skipped and segmentation is done using GT, this difference will dominate the overall speed of the word recognition system. Even though slant and skew correction are not always necessary, segmentation is mandatory and still takes almost a second for each word. Hence, there is only a moderate difference of the costs of the classification methods when integrated into the current word recognition system, as shown in Table 6.

Since word correction on word level is a vital feature but requires calculation of the Levenstein distance *δ* from a recognized word to the complete vocabulary, we also measure its computational time. We got 0.03s±1.6e-3s per word using the moderate vocabulary $V_{5k}$ and 0.40s±0.01s using $V_{50k}$.

Altogether, ∼1 s/word seems to slow in the case that not only single words but complete text pages should be processed. Even using the fast SVM-based or a simplified ASM-based classification, segmentation will remain the bottleneck. However, the segmentation method can be accelerated by approximating minor functions as the baseline detection. Performing segmentation on images of reduced size—by deleting the margins and scaling the cropped image—is an effective strategy, too (but would require to estimate the allowed scaling factor and to transform the segmentation result). It is also known, that implementations in C/C++ are often faster than MATLAB functions. We assume, that about 0.1 s to 0.2 s per word could be achieved with the mentioned improvements. GPU- or FPGA-based implementation have not been considered yet.

## 5. Conclusions and Future Work

We have presented an efficient approach to synthesize Arabic handwritten words and text pages from Unicode. The usage of online sample and Active Shape Model based glyphs and optional manipulations of the character shape allow to generate various images for a given Unicode string to cover the variability of human handwritings. Furthermore, we proposed a technique to create Arabic pseudo-Unicode text, based on the 50,000 most common Arabic words regarding their frequencies. Then, synthetic word databases are created from this pseudo-texts, which are used for the experiments to validate automatic, segmentation based Arabic handwriting recognition. It has been shown that the proposed Active Shape Model based character recognition approach is more suitable than the state of the art Support Vector Machine based classification when being part of a word recognition system. Furthermore, we have shown, that error correction—using a vocabulary of up to 50,000 valid words—clearly improves the word recognition accuracy.

In our future work we are going to investigate methods to segment handwritten text paragraphs into lines and words. Then, in combination with the proposed word recognition approach, synthesized Arabic handwritten text paragraphs shall be utilized to evaluate achievable usage of document categorization. We also going to use the proposed pattern recognition related system for online interpretation of user and machine interactive sign language.

## Figures and Tables

**Table 1:** Arabic Alphabet, Naskh style.

	i	e	m	b		i	e	m	b
Alif	ﺍ	ﺎ			Dhad	ﺽ	ﺾ	ﻀ	ﺿ
Ba	ب	ﺐ	ﺒ	ﺑ	Taa	ط	ﻂ	ﻄ	ﻃ
Ta	ت	ﺖ	ﺘ	ﺗ	Dha	ظ	ﻆ	ﻈ	ﻇ
Tha	ث	ﺚ	ﺜ	ﺛ	Ayn	ع	ﻊ	ﻌ	ﻋ
Jim	ج	ﺞ	ﺠ	ﺟ	Ghayn	غ	ﻎ	ﻐ	ﻏ
Ha	ح	ﺢ	ﺤ	ﺣ	Fa	ف	ﻒ	ﻔ	ﻓ
Kha	خ	ﺦ	ﺨ	ﺧ	Qaf	ق	ﻖ	ﻘ	ﻗ
Dal	د	ﺪ			Kaf	ك	ﻚ	ﻜ	ﻛ
Thal	ذ	ﺬ			Lam	ل	ﻞ	ﻠ	ﻟ
Ra	ر	ﺮ			Mim	م	ﻢ	ﻤ	ﻣ
Zai	ز	ﺰ			Nun	ن	ﻦ	ﻨ	ﻧ
Sin	س	ﺲ	ﺴ	ﺳ	He	ه	ﻪ	ﻬ	ﻫ
Shin	ش	ﺶ	ﺸ	ﺷ	Waw	و	ﻮ		
Sad	ص	ﺺ	ﺼ	ﺻ	Ya	ي	ﻲ	ﻴ	ﻳ

**Table 2:** Validation of the Segmentation Method.

Database	IESK-arDB	IESK-arDB-Syn	$V_{5k}$
Number of Word Samples	2540	9000	8000
Error per word	1.67 ± 0.13	1.74±0.024	0.96 ± 0.019
Error per letter	0.35 ± 0.03	0.34 ± 5 × 10${}^{-3}$	0.27 ± 6.16 × 10${}^{-3}$
Over segmentation (per word)	0.80 ± 0.1	0.86 ± 6 × 10${}^{-3}$	0.41 ± 7.41 × 10${}^{-3}$
Under segmentation (per word)	0.90 ± 0.07	0.88 ± 0.019	0.55 ± 0.013
Perfect segmentation (per word)	0.17 ± 2.5 × 10${}^{-3}$	0.13 ± 7 × 10${}^{-3}$	0.35 ± 8.1 × 10${}^{-3}$

**Table 3:** Quality of writer independent SVM classification, ${}^{*}$ values in () are weighted according class frequency.

Character Form	i	e	m	b
average Precision	90(96)${}^{*}$	77.6(87.2)	89.3(82.3)	92.5(89.7)
average Recall	89.2(95.9)	75.8(89.6)	87.(79.6)	91.1(85.7)
F-score	88.9(95.6)	75.5(87.1)	87.6(80.6)	91.4(87.2)

**Table 4:** Direct ($A_{w}^{\prime }$) and improved ($A_{w}$) Word Recognition Accuracy (using $V_{5k}$).

Rank	ASM	ASM${}_{Gt}$	SVM	SVM${}_{Gt}$
1	49 ±0.67	74 ± 1.7	34 ± 1.7	67 ± 0.0
2	53 ± 0.67	78 ± 0.33	39 ± 0.0	75 ± 2.0
⋮	⋮	⋮	⋮	⋮
10	63 ± 1.3	86 ± 0.67	50 ± 3.0	83 ± 1.0
Fails	37 ± 1.3	14 ± 0.67	50 ± 3.0	17 ± 1.0
$\overset{-}{\delta }$	1.6 ± 0.077	0.69 ± 0.043	2.0 ± 0.16	0.98 ± 0.12
$A_{w}$	49 ± 0.67	74 ± 1.7	34 ± 1.7	67 ± 0.0
$A_{w}^{\prime }$	27 ± 0.33	54 ± 0.1	13 ± 0.67	38 ± 4.7

**Table 5:** Examples of Word Errors.

Original	$\dot{w_{V_{i}}}|\;\delta_{=1}$	$\dot{w_{V_{i}}}|\;\delta_{=2}$	$\dot{w_{V_{i}}}|\;\delta_{=3}$	$\dot{w_{V_{i}}}|\;\delta_{\mathrm{Sim}}$	$\dot{w_{V_{i}}}|\;\delta_{\mathrm{Real}}$
نستطيع	نسدطيع	نلستطي	كستتطيع	فزهطيع	نستﺽيو
ﺍن	ﺍ		سلي	ﺍن	ﺍن
كﺍن	كﺍزن	ﺍطن	صكن	صذن	كﺍن
ﺍلجسم	حﺍلجسم	ﺍلجسمسم	طلةسم	ﺍسسم	ﺍلبسم
رغم	رزغم	ررغم	ت	فجت	غغم
من	ون	ص	كن	من	من

**Table 6:** Computational Time per word.

Segmentation	Classification		s/word
(&Preprocessing) ${}^{*}$	SVM	ASM	
	x		0.06 ± 8.8 × 10^−3^
		x	0.72±0.28
x	x		0.83±0.57 $\left(0.97\pm 0.69\right){\;}^{*}$
x		x	1.31±0.75 $\left(1.51\pm 0.86\right){\;}^{*}$

${}^{*}$ Preprocessing time.
